# Retraction notice to “Global impacts of pre- and post-COVID-19 pandemic: Focus on socio-economic consequences” [Sens. Int. 1 (2020) 100042]

**DOI:** 10.1016/j.sintl.2026.100376

**Published:** 2026-05-01

**Authors:** NT Pramathesh Mishra, Sabya Sachi Das, Shalini Yadav, Wasim Khan, Mohd Afzal, Abdullah Alarifi, El-Refaie Kenawy, Mohammed Tahir Ansari, Md Saquib Hasnain, Amit Kumar Nayak

**Affiliations:** a Department of Pharmacy, Mahatma Gandhi Institute of Pharmacy, Lucknow, 227101, Uttar Pradesh, India; b Department of Pharmaceutical Sciences and Technology, Birla Institute of Technology, Mesra, Ranchi, 835 215, Jharkhand, India; c Abha Biotechnology, Noida, 201301, Uttar Pradesh, India; d Department of Pharmaceutics, Javitri Institute of Medical Sciences &Pharmacy Division, Lucknow, 227302, Uttar Pradesh, India; e Catalytic Chemistry Chair, Department of Chemistry, College of Science, King Saud University, Riyadh, 11451, Saudi Arabia; f Polymer Research Group, Department of Chemistry, Faculty of Science, Tanta University, Tanta, Egypt; g School of Pharmacy, University of Nottingham Malaysia, Jalan Broga, Semenyih, Selangor, 43500, Malaysia; h Department of Pharmacy, Shri Venkateshwara University, NH-24, Rajabpur, Gajraula, Amroha, 244236, U.P., India; i Department of Pharmaceutics, Seemanta Institute of Pharmaceutical Sciences, Mayurbhanj, 757086, Odisha, India

This article has been retracted: please see Elsevier policy on article withdrawal (https://www.elsevier.com/about/policies-and-standards/article-withdrawal).

This article has been retracted at the request of the Editor.

Post-publication, concerns were raised on PubPeer that the article contained several “tortured phrases” which make some passages hard to parse because they deviate from standard technical terms, as detailed here: https://pubpeer.com/publications/2126AE9C6DF69C0B4408EB87B78C1C?utm_source=Chrome&utm_medium=BrowserExtension&utm_campaign=Chrome.

The journal editor assessed the article and found additional phrases that deviate from standard technical terms. The authors were requested to explain the use of these passages of text but were unable to do so. The Editor has lost confidence in the findings of the article and has determined that it should be retracted.

The investigation also identified a change in authorship between the original submission and the revised version of this paper. During revision, the author (Kakarla Raghava Reddy) was added to the revised paper without explanation and without exceptional approval by the journal editor, which is contrary to the journal policy on changes to authorship.

The authors disagree with the retraction and dispute the grounds for it.

